# Effect of Discontinuation of Fluoride Intake from Water and Toothpaste on Urinary Excretion in Young Children

**DOI:** 10.3390/ijerph8062132

**Published:** 2011-06-10

**Authors:** Carolina C. Martins, Saul M. Paiva, Jaime A. Cury

**Affiliations:** 1 Department of Paediatric Dentistry and Orthodontics, School of Dentistry, Federal University of Minas Gerais, Avenida Antônio Carlos 6627, Belo Horizonte, MG, Brazil; E-Mail: carolcm10@hotmail.com; 2 Department of Biochemistry, Piracicaba Dental School, University of Campinas, Avenida Limeira 901, Piracicaba, SP, Brazil; E-Mail: jcury@fop.unicamp.br

**Keywords:** fluoride, toothpaste, urine, drinking water

## Abstract

As there is no homeostatic mechanism for maintaining circulating fluoride (F) in the human body, the concentration may decrease and increase again when intake is interrupted and re-started. The present study prospectively evaluated this process in children exposed to F intake from water and toothpaste, using F in urine as a biomarker. Eleven children from Ibiá, Brazil (with sub-optimally fluoridated water supply) aged two to four years who regularly used fluoridated toothpaste (1,100 ppm F) took part in the study. Twenty-four-hour urine was collected at baseline (Day 0, F exposure from water and toothpaste) as well as after the interruption of fluoride intake from water and dentifrice (Days 1 to 28) (F interruption) and after fluoride intake from these sources had been re-established (Days 29 to 34) (F re-exposure). Urinary volume was measured, fluoride concentration was determined and the amount of fluoride excreted was calculated and expressed in mg F/day. Urinary fluoride excretion (UFE) during the periods of fluoride exposure, interruption and re-exposure was analyzed using the Wilcoxon test. Mean UFE was 0.25 mg F/day (SD: 0.15) at baseline, dropped to a mean of 0.14 mg F/day during F interruption (SD: 0.07; range: 0.11 to 0.17 mg F/day) and rose to 0.21 (SD: 0.09) and 0.19 (SD: 0.08) following F re-exposure. The difference between baseline UFE and the period of F interruption was statistically significant (p < 0.05), while the difference between baseline and the period of F re-exposure was non-significant (p > 0.05). The findings suggest that circulating F in the body of young children rapidly decreases in the first 24 hours and again increases very fast after discontinuation and re-exposure of F from water and toothpaste.

## 1. Introduction

There has been an increase in the search for biomarkers for monitoring different sources of fluoride (F) intake [1], including fingernails [2,3] and urine [4]. Among the F intake sources, drinking water and toothpastes are considered risk factors of fluorosis [5], although the real contribution of each source to the development of fluorosis is not clear and a dose-response effect has not yet been established [6].

The use of urine as potential biomarker of F intake from diet and fluoridated toothpastes has been investigated [7–12]. These studies have an observational design and evaluate F intake from water and toothpastes at a single point in time, with no experimental data and no follow up of the individuals. Thus, there is a lack of follow-up studies aimed at evaluating the variation in urinary fluoride excretion (UFE) when F is discontinued. Knowledge on the role of urine as a biomarker of F is important to providing data on the amount of F ingested by children at ages of risk for the development of dental fluorosis.

UFE under different concentrations of F in water and toothpaste over time should also be evaluated. There is insufficient scientific evidence to indicate how many days it takes UFE to decrease and remain stable after F intake is discontinued. Moreover, there is a lack of methodological standardization in studies investigating UFE following the discontinuation of F intake. Such studies have comprised individuals in different age groups [13,14], with different sources of F, such as in supplements [14,15] or milk [15], and different F concentrations [13]. Considering the lack of scientific evidence on the effect of the discontinuation of F from water and toothpaste, it is important to investigate the release of F retained following the discontinuation of intake and the effect after F re-exposure under controlled conditions, using urine as a biomarker. Thus, the aim of the present study was to carry out a prospective investigation into the effect of the discontinuation of F intake from the water supply and fluoridated toothpaste.

## 2. Experimental Section

The present study received approval from the Ethics Committee of the Federal University of Minas Gerais (project number: 279/07). All parents/guardians received information regarding the objectives of the study and signed terms of informed consent.

### 2.1. Subjects

Eleven healthy children (six girls and five boys) aged two to four years (mean age: 43.9 months; 34 to 56 months) participated in the present prospective study in May and June 2008. The investigation took place in the city of Ibiá, MG, Brazil. All children had lived in Ibiá since birth. Water samples were collected from the public water supply, which was artificially fluoridated by the municipal water treatment system and was determined to be sub-optimally fluoridated.

Children were recruited at a private day care centre. The following were the inclusion criteria: good systemic health status; good oral health status; age between two to four years; absence of medications or fluoridated supplements; and very high parent compliance with the study protocol. Parents were interviewed regarding their children’s overall health, tooth brushing habits, diet and use of fluoridated supplements. The main fluoride sources were fluoridated toothpaste and public drinking water. All children regularly used a Tandy^®^ toothpaste, which is a fruit flavoured children’s toothpaste with a fluoride concentration declared by the manufacturer of 1,100 ppm F, sodium fluoride and silica as the abrasive (Tandy^®^, Colgate/Palmolive Ind. Ltda, São Bernardo do Campo, SP, Brazil). All children were weighed prior to the beginning of the study (mean weight: 17.6 ± 4.74 kg).

### 2.2. Experimental Design

**Figure 1.**
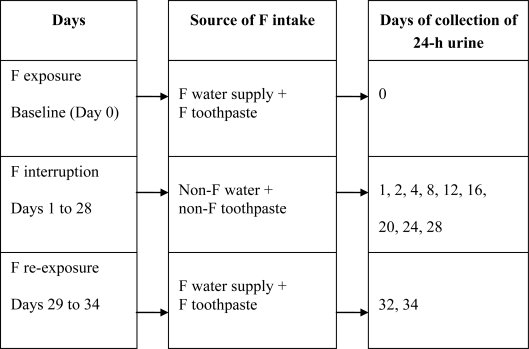
Illustration of experimental design.

Twenty-four-hour urine was collected to determine urinary fluoride excretion (UFE) under normal conditions (baseline — Day 0). The children then refrained from fluoride intake from water and toothpaste use until Day 28 (F interruption). Twenty-four-hour urine was collected during F interruption on Days 1, 2, 4, 8, 12, 16, 20, 24 and 28 (Figure 1).

From Days 1 to 28, the children were supplied with de-ionized water prepared for the study at a local laboratory (Laboratório Vita Center, Ibiá, MG, Brazil) and the families were given a daily supply of five-litre bottles. The de-ionized water was analyzed for fluoride content at the Piracicaba Dental School of the University of Campinas (Brazil) and had a mean concentration <0.01 ppm F. The parents were carefully instructed that all drinks, juices, food, soups and powdered milk were to be prepared with this water. It was stressed that the de-ionized water was to be the only water that the children would consume throughout this phase of the experiment. Parents were instructed that children should avoid teas and any kind of seafood, as some of these items can have considerable F concentrations [16].

The children were also supplied with a non-fluoridated toothpaste (Malvatri Kids Baby^®^, Laboratório Daudt Oliveira Ltda, Rio de Janeiro, RJ, Brazil) and a children's toothbrush. The parents were instructed that the children were to use only this toothpaste during the 28-day period at a frequency of three times per day. This frequency was intended to introduce a routine for oral hygiene in the children. No other toothpaste or other topical fluoride was to be used during this phase of the experiment.

All volunteers were recruited from the same day care centre in order to facilitate the water control. The day care centre was given a daily supply of de-ionized water. The children enrolled in the study had day care in the afternoon. The afternoon snack was prepared at home. The most common snacks brought by children were: chocolate milk, yogurts, manufactured or homemade biscuits, juice, bananas and apples. However, the fluoride content of the snacks was not evaluated. After recreation, the children usually brushed their teeth. Therefore, the day care staff members received the non-fluoridated toothpaste and were instructed to use only this toothpaste during this phase of the experiment. The experiment was carefully supervised by one of the researchers, who was present at the day care centre every day so the research protocol could be monitored. The deionized water was delivered every morning by the same researcher, at which time the instructions were reinforced.

On Day 29, the parents were instructed to re-establish the previous fluoride conditions (F re-exposure). The non-fluoridated water regimen was suspended and the parents received a fluoridated toothpaste to be used by the children when brushing (Tandy^®^, 1,100 ppm F, Colgate/Palmolive Ind. Ltda, São Bernardo do Campo, SP, Brazil). The day care staff received the same toothpaste and the drinking water went back to being from the public water supply. Twenty-four-hour urine was collected under fluoridated conditions on Days 32 and 34.

### 2.3. Collection of 24-Hour Urine

Urine was collected in 24-h periods. The children were provided with a two-litre plastic collection bottle with a screw top. Parents were instructed with regard to the importance of collecting all urine during the 24-h period. At the day care centre, the urine was collected every time the children asked to go to the bathroom. The bottles were labelled with the children’s names. The parents deposited the urine from the previous day at the day care centre on the scheduled days. The volume collected at home was mixed with the volume collected at the day care centre. The total volume was recorded and approximately 20 mL was placed in a sterile plastic cylinder containing thymol as a preservative (Amphora Farmácia de Manipulação Ltda, Belo Horizonte, MG, Brazil). The samples were frozen until analysis. Fluoride elimination via faeces and sweat was not assessed. The samples were sent to the Piracicaba Dental School of the University of Campinas and analyzed for fluoride content.

### 2.4. Collection of Public Water Supply

Samples from the public water supply were collected for seven days prior to beginning the study. Samples from the public water supply were also collected from Days 29 to 34. All water samples were sent for analysis at the Piracicaba Dental School of the University of Campinas.

### 2.5. Fluoride Analysis

Fluoride was analyzed in duplicate aliquots buffered with TISAB II using an ion-selective electrode (Orion 96-09, Orion Research, Cambridge, MA, USA) and ion analyzer (Orion EA-940), which were previously calibrated with standard F solutions (Orion 940907). Analysis was validated using internal standards and a coefficient variation lower than 3% was considered acceptable.

### 2.6. Data Analysis

Descriptive analysis was carried out. The quantitative variables were tested for normality using the Kolmogorov-Smirnov test. Levene’s test was used to determine the homogeneity of the variance. As the data had non-normal distribution and variance was not homogeneous, the non-parametric Friedman was used to determine statistically significant differences in fluoride urinary excretion values between evaluations; and Wilcoxon test was used to determine statistically significant differences in fluoride urinary excretion day-by-day.

## 3. Results

Mean 24-hour urinary volume from baseline (Day 0) to Day 34 was 398.11, standard deviation (SD) ±231.97 mL (minimum: 296.67, SD ± 172.34 mL; maximum: 471.36, SD ± 353.66 mL). There were no statistically significant differences in mean urinary volume between evaluations (p > 0.05).

**Figure 2.**
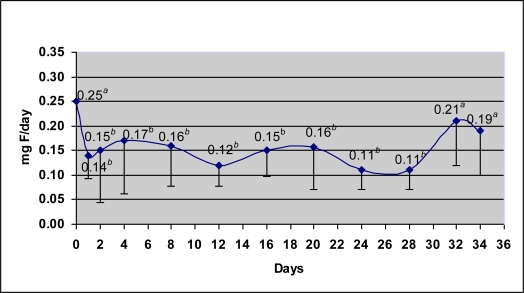
Mean urinary fluoride excretion (mg F/day) at baseline (Day 0), after interruption of fluoride intake from water and toothpaste (Days 1 to 28) and after fluoride intake had been re-established (Days 29 to 34); statistically significant difference in mean UFE between Days 0 to 34 (p = 0.013, Friedman test); means followed by different letters are statistically different (Wilcoxon test; p ≤ 0.05); bars represent standard deviation (n = 11).

Figure 2 displays the mean 24-h urinary fluoride excretion (UFE) over time. At baseline, fluoride excretion was 0.25 mg F/day (SD: 0.15), which rapidly decreased to 0.14 mg F/day on Day 1. Fluoride excretion then varied little through to Day 28, maintaining a mean of 0.14 ± 0.07 mg F/day. UFE rose again on Days 32 (0.21 ± 0.09 mg F/day) and 34 (0.19 ± 0.08 mg F/day), but did not reach the baseline value. The Friedman test revealed a significant difference between Days 0 to 34 (p = 0.013). The Wilcoxon test for paired comparisons was used to verify the differences between paired days. There was a statistically significant difference in UFE between baseline (0.25 mg F/day) and the mean value of the period from Day 1 to 28 (0.14 mg F/day; Wilcoxon test, p < 0.05). The Wilcoxon test also revealed significant differences between baseline and all days following F interruption (p < 0.05, represented in Figure 2 by different letters). However, baseline and day 32 and baseline and day 34 were not statistically different.

Fluoride content in the samples of the public water supply was analyzed for seven days prior to beginning the study. The water was sub-optimally fluoridated (mean: 0.2 ppm F; range 0.17 to 0.2). When the fluoride intake was re-established (Days 29 to 34), the levels of fluoride in the drinking water had rose to a mean average of 0.4 ppm F (range 0.38 to 0.46).

Table 1 displays UFE values at baseline, during the period of F interruption and Days 32 and 34. The values for Days 32 and 34 were adjusted as if the water had maintained a constant level of 0.2 ppm F throughout the entire study. In order to calculate the adjusted values, the study of Paiva *et al*. [17] was used as it was conducted in the same city as the present study (Ibiá). Although in the previous study Ibiá had optimally fluoridated water (0.7, range 0.6–0.8 ppm F) and children from both studies are different, both studies were conducted with a convenience sample of individuals of high socioeconomic status. These adjusted values were 0.17 and 0.16 mg F/day, respectively. As 35% of fluoride intake is from water and 65% is from brushing with a fluoridated toothpaste [17] and considering the positive correlation between fluoride intake and urinary fluoride excretion [9,12], 0.07 mg F/day of the value excreted on Day 32 was from water intake (35% of 0.21 mg F/day = 0.07) and 0.14 mg F was from toothpaste intake (65% of 0.12 mg F/day = 0.14). As the water had twofold more fluoride on Days 32 and 34 (0.4 ppm F) than at baseline (0.2 ppm F), the children would have ingested half the amount of fluoride from water on these days if the content were at 0.2 ppm F and would have excreted half the fluoride from water intake (half of 35%, which is ½ of 0.07 = 0.035 mg F/day). Thus, adding 0.035 mg F/day (excreted due to water intake) to 0.14 mg F/day (excreted due to toothpaste intake), the final adjusted value is 0.17 mg F/day of fluoride excreted on Day 32 if the water had had a F content of 0.2 ppm. The same calculation was made for Day 34. Considering the adjusted values, UFE at baseline (0.25 mg F/day) was significantly different from Days 1 to 28 (0.14 mg F/day) as well as Days 32 (0.17 mg F/day) and 34 (0.19 mg F/day) (p ≤ 0.05) and there were no statistically significant differences between Days 1 to 28 and Days 32 and 34 (p > 0.05).

## 4. Discussion

Twenty-four-hour urine sampling is a reliable method and measures the fluoride content of an entire day. However, it requires a very high degree of family compliance and there is a risk of sample loss. In order to minimize such loss, the families were carefully prepared and selected in order to achieve maximal cooperation. The effort on the part of the parents to cooperate with the study is evidenced by the little variation in mean urinary volume throughout the study (mean: 398.11 ± SD 231.97 mL).

Urinary fluoride excretion at baseline (Day 0) was 0.25 mg F/day (SD: 0.15) and fluoride content in the public water supply was 0.2 ppm, which is close to the values found in the literature on UFE under sub-optimal water fluoride conditions (0.20 to 0.39 mg F/day for water with ≥0.15 to <0.7 ppm F) [8,9,11,12].

The data suggest that urine as a biomarker can rapidly detect variations in F intake and stabilization was achieved in about 24 hours. This is in agreement with the literature, which reports that fluoride is excreted from the body in the hours following its intake from fluoridated salt, fluoridated toothpaste [18] and fluoridated water [19]. When fluoride conditions were re-established, UFE increased again. This finding corroborates the results of a previous study, in which UFE rose from 0.18 to 0.33 mg F/day when children refrained from ingesting fluoride for three days and then received a 0.5-mg F tablet with half glass of water over the subsequent two days [15]. One may speculate that if the period of F interruption were longer, UFE might continue to decline over time, albeit very slowly. A previous study reported that UFE continued to exhibit a slight decrease even after 113 weeks; however, subjects had been chronically exposed to an above-optimal water supply (8.0 ppm F) and the water was de-fluoridated to an optimal level (0.7 ppm F) [13].

The results may have been different if the experiment had been conducted with adults, as fluoride retention (percentage of absorbed fluoride), extrarenal clearance, skeletal uptake [20] and fluoride removal from the plasma is generally greater in children than adults [21,22]. This may explain the rapid decrease in fluoride excretion in the first 24 hours. A negative balance (when fluoride intake is less than fluoride excretion) may indicate that skeletal and perhaps dental stocks of fluoride are being depleted [22]. However, the rapid decrease in fluoride excretion in the present study did not indicate that skeletal and dental stocks were totally depleted, as UFE continued to be excreted throughout the period of F interruption, thereby indicating that exchanges between mineralized tissues and plasma continued to occur. Besides the F reservoir in the bones, UFE during the period of F interruption never achieved values near 0 due to the natural F content in meals [23].

When the fluoride conditions were re-established, UFE increased, but neither reached the baseline value nor remained stable, although the fluoride content in the water had increased twofold (to 0.4 ppm F). One hypothesis is that some mothers may have forgotten to use the fluoridated toothpaste at the recommended frequency. Another explanation is that young children may take longer to have stabilization of fluoride excretion after the onset of F intake. Two studies have evaluated the onset of salt and water fluoridation in two communities with no previous history of fluoride in the drinking water [24] or salt [25]. For children, it took 20 months to reach a stabilization on UFE after the onset of salt fluoridation [25] and approximately three years after the onset of water fluoridation, while the adults had stabilized in one week [24].

The children in Ibiá were found to be exposed to a F dose of 0.08 mg F/Kg/day from water and toothpaste [17]. Multiplied by the mean body weight of the children in the present study (17.59 ± 4.74 Kg), mean F intake was about 1.41 mg F/day. UFE was 0.25 mg F/day at baseline, which corresponds to 17.7% excretion of total F intake. Considering 10% excretion through faeces [26,27], about 72.3% was retained by the children [100% – (17.7% + 10%) = 72.3%]. The higher UFE values reported in the literature (30.0% to 80.0%) [8–10,12] and mean retention of 50 to 55% [8,12] have been suggested to be inaccurate for lower F intake and actively growing young children, whose bone turnover is higher [20], as in the case of the children in the present study.

The present study has some limitations that should be considered when interpreting the results. Due to the complexity of the experimental design, a convenience sample was used and it was not possible to conduct the study with a larger sample. A single baseline measurement was performed and there was limited follow up after restarting the usual fluoride intake from water and toothpaste. Unfortunately, it was not possible to continue the study until stabilizing fluoride excretion, as the study was designed to last only one month in order to determine the decrease in fluoride excretion. The study was conducted under conditions of sub-optimally fluoridated drinking water and the fluoride content in the water varied throughout the study period. Variation of fluoride content in water was not expected, and the only explanation for this variation was the negligence of the water treatment system. The results may have been different if the water had been optimally fluoridated. The results may also have been different if the study had been conducted in the summer, as fluoride intake by children may vary depending on the season [28]. In Brazil, May and June are late autumn months, when children may drink less water than in summer. Further studies should be performed to evaluate fluoride excretion with an optimally fluoridated water supply associated to fluoridated toothpastes.

## 5. Conclusions

The findings of the present study suggest that circulating F in the body of young children rapidly decreases in the first 24 hours after discontinuation of F from water and toothpaste and increases very fast again after re-exposure of F from the same sources.

## Figures and Tables

**Table 1.** Mean (±SD; n = 11) amount of fluoride (mg F/day) in urine of children from Days 0 to 34 and adjusted values based on fluoride intake.

**Days**	**Source of F intake**	**mg F/day**	
		**Found**	**Adjusted**
**F exposure (0)**	Water + Toothpaste	0.25 ± 0.15 ^a^	0.25 ± 0.15 ^a^
**F interruption (1 to 28)**	None	0.14 ± 0.02 ^b^	0.14 ± 0.02 ^b^
**F re-exposure (32)**	Water + Toothpaste	0.21 ± 0.09 ^a^	0.17 ± 0.07*^b^
**(34)**	Water + Toothpaste	0.19 ± 0.08 ^a,b^	0.16 ± 0.06*^b^

* Means adjusted considering water at 0.2 ppm F;

a,b Means (within columns) followed by different letters are statistically different (Wilcoxon test; p ≤ 0.05).
